# Visual Ability and Searching Behavior of Adult *Laricobius nigrinus*, a Hemlock Woolly Adelgid Predator

**DOI:** 10.1673/031.011.11101

**Published:** 2011-08-29

**Authors:** D.L. Mausel, S.M. Salom, L.T. Kok

**Affiliations:** ^1^ Virginia Tech, Department of Entomology, 216A Price Hall MC 03 19 Blacksburg, VA 24061-0002; ^2^ Current address: University of Massachusetts, Department of Plant, Soil and Insect Sciences Agricultural Engineering Bldg., 250 Natural Resources Rd., Amherst, MA 01003-9295

**Keywords:** biological control, hemlock woolly adelgid, monitoring, predation, *Tsuga canadensis*, visual cues

## Abstract

Very little is known about the searching behavior and sensory cues that *Laricobius* spp. (Coleoptera: Derodontidae) predators use to locate suitable habitats and prey, which limits our ability to collect and monitor them for classical biological control of adelgids (Hemiptera: Adelgidae). The aim of this study was to examine the visual ability and the searching behavior of newly emerged *L. nigrinus* Fender, a host-specific predator of the hemlock woolly adelgid, *Adelges tsugae* Annand (Hemiptera: Phylloxeroidea: Adelgidae). In a laboratory bioassay, individual adults attempting to locate an uninfested eastern hemlock seedling under either light or dark conditions were observed in an arena. In another bioassay, individual adults searching for prey on hemlock seedlings (infested or uninfested) were continuously video-recorded. Beetles located and began climbing the seedling stem in light significantly more than in dark, indicating that vision is an important sensory modality. Our primary finding was that searching behavior of *L. nigrinus*, as in most species, was related to food abundance. Beetles did not fly in the presence of high *A. tsugae* densities and flew when *A. tsugae* was absent, which agrees with observed aggregations of beetles on heavily infested trees in the field. At close range of prey, slow crawling and frequent turning suggest the use of non-visual cues such as olfaction and contact chemoreception. Based on the beetles' visual ability to locate tree stems and their climbing behavior, a bole trap may be an effective collection and monitoring tool.

## Introduction

All known tooth-necked fungus beetles (Coleoptera: Derodontidae) in the genus *Laricobius* Rosenhauer are arboreal predators of adelgids (Hemiptera: Adelgidae), a prey that feeds on sap or stored starches of trees in the Pinaceae family (Blackman and Eastop 1994). The Derodontidae have rarely garnered much attention beyond taxonomists. However, considerable autecological research has been conducted on *L. nigrinus* Fender because of its potential as a classical biological control agent of the hemlock woolly adelgid, *Adelges tsugae* Annand.

Native to the western United States and Canada, *L. nigrinus* is a host-specific predator of *A. tsugae* (Zilahi-Balogh et al. 2002), which is typically at low densities and rarely a pest on western hemlock, *Tsuga heterophylla* (Rafinesque) Sargent (Pinales: Pinaceae) (Collman 1972; Furniss and Carolin 1977). In the eastern United States, a Japanese population of *A. tsugae* was accidentally introduced (Havill et al. 2006) and has become the most important pest of eastern hemlock, *T. canadensis* (L.) Carrière. To evaluate *L. nigrinus* as a biological control agent, life-history studies were conducted showing that they could be effective (Zilahi-Balogh et al. 2003a; Zilahi-Balogh et al. 2003b; Zilahi-Balogh et al. 2003c; Lamb et al. 2005; Zilahi-Balogh et al. 2006). *L. nigrinus* were released in the eastern United States and became established (Mausel et al. 2010).

*L. nigrinus* adults feed in fall, winter, and early spring on their primary prey, *A. tsugae*, which are sessile at the base of needles. Females lay eggs in adelgid ovisacs (egg clusters) from late winter to early spring, and eclosing beetle larvae are encircled by eggs, their preferred prey stage. Late instars may search twigs for eggs or nymphs if all the eggs in an ovisac are eaten, as is the case for *L. erichsonii* (Franz 1958). In spring, fourth instar larvae drop to the forest floor and pupate in earthen cells. Adults eclose and enter aestival diapause (Zilahi-Balogh et al. 2003a; Lamb et al. 2007). In fall, adults emerge from the forest floor after several months of non-feeding. As such, beetles must quickly locate hemlock trees and *A. tsugae*, or starve.

Very little is known about *Laricobius* prey searching behavior and, for practical purposes, research on *L. nigrinus* is needed to guide development of a lure and trap system to improve collection and monitoring. A description and quantification of adult *L. nigrinus* behavior in Petri dishes (Flowers et al. 2007), physiological description of the antennae (Broeckling and Salom 2003a), and hemlock odor bioassays (Broeckling and Salom 2002) indicate that olfaction plays a role in prey searching and detection. The aim of this study was to examine another sensory modality--vision--in prey searching by *L. nigrinus*. In addition, the activity of newly emerged adults was described and quantified on *A. tsugae*-infested and uninfested hemlock seedlings to provide a natural context and spatial scale during a critical phase of their prey searching behavior.

## Materials and Methods

### Insects

Adult *L. nigrinus* used in the following bioassays were laboratory-reared progeny from adults collected near Victoria, British Columbia, Canada (48.48° N, 123.36° W) in late winter. Rearing conditions followed standardized protocols (Lamb et al. 2005).

The following fall, after aestival diapause, newly emerged adults were individually held in Petri dishes on wet filter paper and held at 15° C and 12:12 L:D for up to 24 h before each bioassay. Eastern hemlock seedlings with high densities of *A. tsugae* (75% of needle cushions infested) were collected from locally infested forests in Giles Co., Virginia.

### Visual cue bioassay

The experimental arena was a soil filled pot with a 177 cm^2^ surface area (15 cm diameter) in a climate controlled room at 9.7–12.9° C and 42–78% RH. The arena size was deemed adequate given the small size of the beetles (2–3 mm length). An uninfested hemlock seedling (40 cm tall, 5 years old, and 0.5 cm diameter at the root crown) was positioned in the middle of the arena. Although beetles also encounter saplings and mature trees in the field, we did not attempt to mimic these trees in the laboratory. A border of white paper covered in fluon was placed along the perimeter of the arena and the arena was covered in beige-colored sand. Individual *L. nigrinus* adults were placed in the arena (7 cm from the seedling) and recorded as locating the seedling or not during an arbitrary 10-minute time period under either light (25W soft-white light bulb to mimic daylight) or dark (25W red light bulb to mimic night) conditions (*n* = 20). Beetles were randomly assigned to the light treatment and not reused. Beetle sex was not determined. The same bioassay was repeated in winter to confirm results of the fall experiment. The beetles in winter had fed on *A. tsugae* for two months in rearing containers.

### Prey searching behavior bioassay

Prey searching behavior was studied on *A. tsugae*-infested and uninfested hemlock seedlings (*n* = 10) in the laboratory using the same experimental arena, similarly sized seedlings, and environmental conditions described previously. First, an ad libitum observation of 13 beetles on the seedlings was conducted to catalogue behaviors. Nine behaviors were defined for examination during the bioassay (Table 1). Individual beetles were placed on the arena surface with either an *A. tsugae*-infested or uninfested seedling in a 0.45 m^3^ muslin cage under white light. They were continuously recorded with a Panasonic Digital Palmcorder Model PVGS35 (Panasonic, www.panasonic.com) that was linked to a desktop computer to transfer, archive, and watch the video (Flowers et al. 2007). The camera was held by hand and adjusted when plant parts obscured the beetles. Video recording started when beetles contacted the stem and ended when they began feeding on *A. tsugae* (i.e., prey searching was successful), flew off the seedling, or 30 min had expired. From the footage, the frequency of each behavior (bouts), elapsed time until each behavior began (latency) and the total time length of each behavior, when applicable, were recorded in seconds. Beetles were randomly assigned the seedling treatment and not reused. Beetle sex was determined after each bioassay by protraction of the genitals.

### Data analyses

The visual cue bioassay data were arranged in a 2 × 2 contingency table to test if the proportion of beetles locating trees in either light or dark was similar (using Yate's correction for continuity). To determine if results from the fall and winter bioassays were homogenous, a heterogeneity chi-square analysis was conducted (using Pearson's Chisquare).

For the prey searching behavior bioassay, the bouts, latency, and total time length for each behavior, when applicable, were compared between *A. tsugae*-infested and uninfested seedlings by non-parametric Mann-Whitney *U* tests (0.05 significance level). All data are presented as standard error of the mean. In addition, the number of times a beetle switched from one behavior to another was tallied and behavioral sequence diagrams were created from the pooled activity of ten individual beetles on each treatment using Microsoft PowerPoint. Arrows were used in the diagrams to indicate the sequence of behaviors; their font size indicates the relative frequency of behavioral flow.

**Figure 1.**
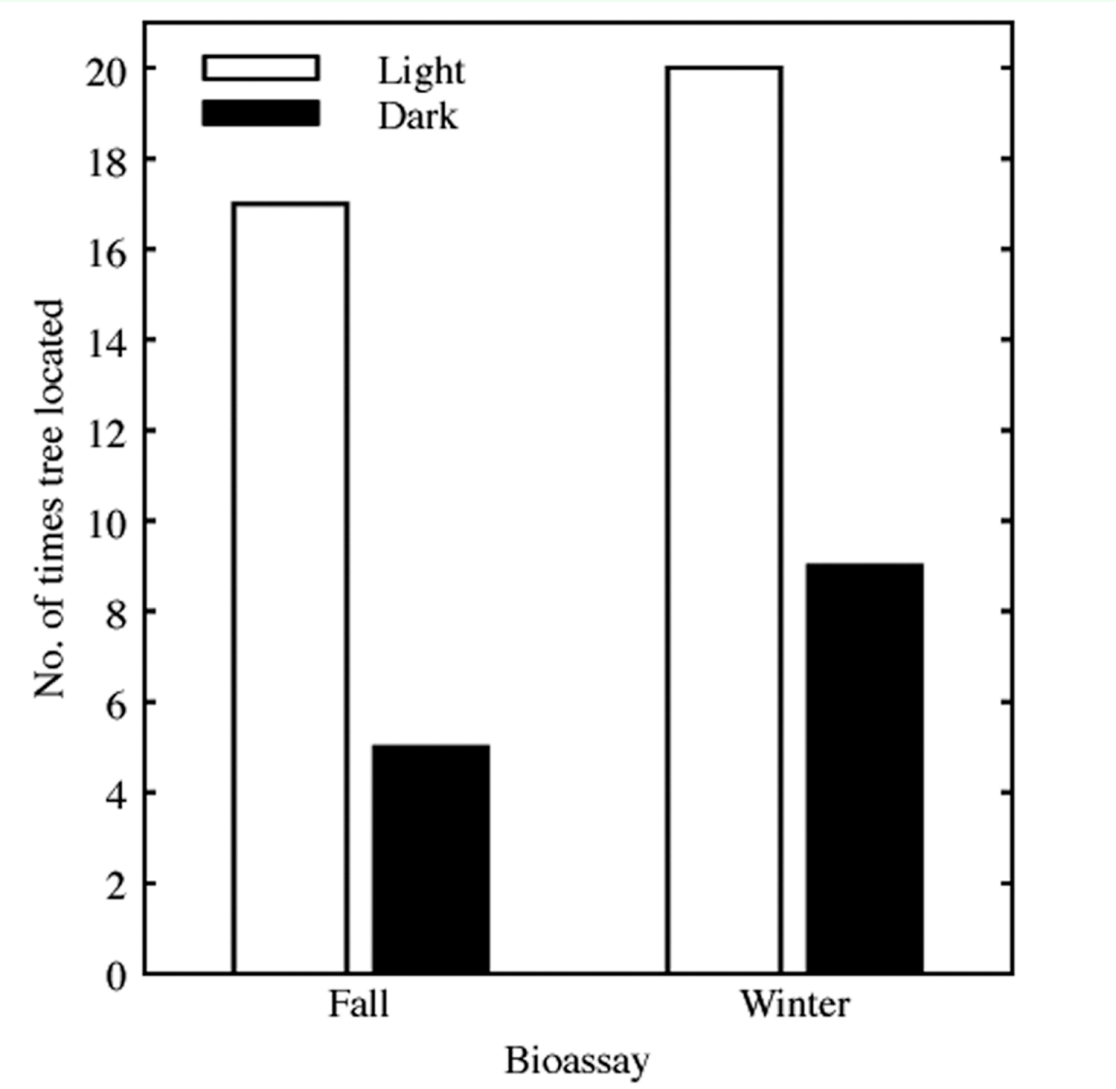
The frequency (out of 20 replications) with which individual *Laricobius nigrinus* adults located an eastern hemlock seedling in an experimental arena in either light or dark conditions. Bioassays were conducted in fall and winter. High quality figures are available online

## Results

### Visual cue bioassay

In fall, light had an effect on the probability of *L. nigrinus* adults locating trees (χ_y_^2^ = 12.2, df = 1, *P* = 0.0005; Figure 1). Beetles were 3.4 times as likely to find trees in light than in dark (Video 1). In winter, light also had an effect on the probability of beetles locating trees (χ_y_^2^ = 12.5, df = 1, *P* = 0.0004; Figure 1). Beetles were 2.2 times as likely to find trees in light than in dark. There was no difference between the fall and winter bioassays (Heterogeneity χ^2^ = 1.11, df = 1, *P* = 0.3).

**Figure 2.**
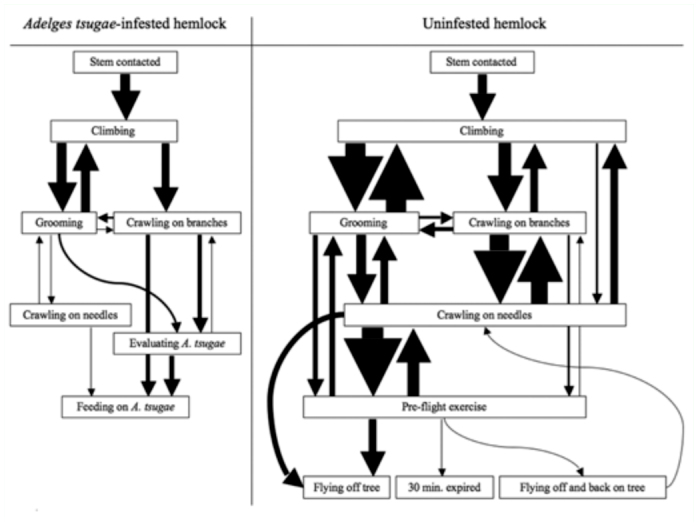
Sequence diagram of newly emerged *Laricobius nigrinus* adult behavior while searching for their prey, *Adelges tsugae*, on either infested or uninfested hemlock seedlings in the laboratory. Arrow font sizes indicate the relative frequency of behavioral flow pooled from the activity of ten individual beetles in each treatment. High quality figures are available online

### Prey searching behavior bioassay

In total, 55.7 min of adult *L. nigrinus* behavioral footage on hemlock seedlings were analyzed to the nearest second. Each treatment had 1:1 (male:female) sex ratios. On the *A. tsugae-*infested seedlings, 100% of the beetles ultimately fed (Figure 2). In general, beetles climbed, groomed, crawled on branches, evaluated *A. tsugae*, and then fed. The cycle of grooming and climbing was common, and beetles less frequently cycled between grooming and crawling on branches, crawling on branches and evaluating *A. tsugae*, and grooming and crawling on needles. Beetles spent the most time climbing, grooming, crawling on branches, crawling on needles, and evaluating *A. tsugae* in descending order (Table 2). Crawling on needles only occurred once. Beetles did not engage in pre-flight exercise or flight and, on average, began evaluating *A. tsugae* in 3.7 min and feeding in 5.6 min. Evaluation of *A. tsugae* was uncommon and was observed approximately once for every two beetles tested (Video 2).

On uninfested seedlings, 90% of the beetles ultimately flew off, and one individual searched until the 30 min bioassay ended (Figure 2). Several behavioral cycles occurred and the prevailing sequence of searching behavior was complex. Common cycles were climbing and grooming, climbing and crawling on branches, grooming and crawling on needles, crawling on branches and crawling on needles, and crawling on needles and pre-flight exercise. Less common cycles were climbing and crawling on needles, grooming and crawling on branches, grooming and pre-flight exercise, and crawling on branches and pre-flight exercise. Beetles spent the most time grooming, climbing, crawling on needles, crawling on branches, and in pre-flight exercise in descending order (Table 2). Pre-flight exercise occurred three times on average and for 56 s per individual. Flight exercise began on average in 11.4 min and flight off the seedling in 15.4 min (Video 3). Flight off and back on the seedlings only occurred once.

Beetles had significantly more bouts of climbing, grooming, crawling on branches, and crawling on needles on uninfested seedlings compared with *A. tsugae*-infested seedlings (Table 2). There was also significantly more time spent climbing and crawling on needles on uninfested than infested seedlings. There were no significant differences in the time spent or latency of grooming or crawling on branches on uninfested compared with infested seedlings.

**Table 1.**
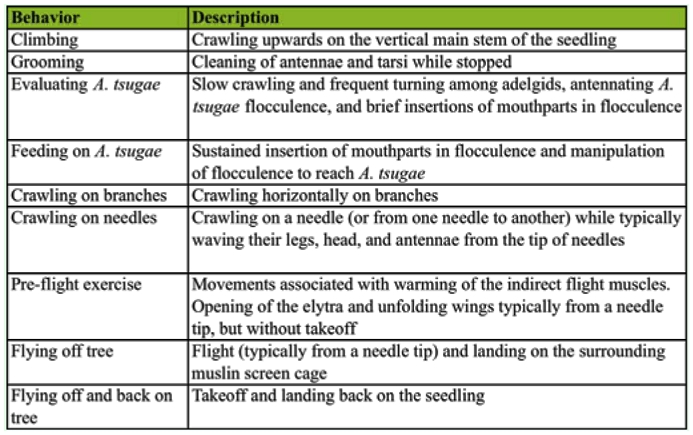
*Laricobius nigrinus* behaviors analyzed from the continuous video footage of prey searching on either *Adelges tsugae*-infested or uninfested seedlings in the laboratory.

## Discussion

Following fall emergence of aestivating adults, the influence of olfactory or visual cues on *L. nigrinus* likely varies depending on spatial scale. Olfaction may be used by *L. nigrinus* (Broeckling and Salom 2002; Broeckling and Salom 2003a) to locate trees from large distances via volatile emissions from *A. tsugae*-infested trees (Broeckling and Salom 2003b), although this needs experimental confirmation. The frequency at which *L. nigrinus* is found on isolated infested trees in its native range underscores the capability of beetles to perceive *A. tsugae* at large distances (D. Mausel, Pers. obs.). Use of vision by *L. nigrinus*, at least at close range, was not a surprising result in the visual cue bioassay as insectivores typically rely on more than one sensory modality (Goyer et al. 2004; Laubertie et al. 2006). For example, tiger beetles (Coleoptera: Cicindelidae) are exceptional visual predators but can capture prey in darkness using other senses (Riggins and Hoback 2005). In darkness, *L. nigrinus* typically searched the arena by continuously and randomly turning between straight courses. This behavior is described as “ranging,” because there was little spatial information (Jander 1975). Locating the stem by chance took the beetles a relatively long time, but on occasion beetles perceived and located it when they were within a centimeter. Conversely, in light beetles took a straight course to the seedling stem relatively quickly (Video 1). This behavior is described as the “approach” and conclusion of object orientation searching as beetles were visually guided to the precise location of the stem (Jander 1975).

**Table 2.**
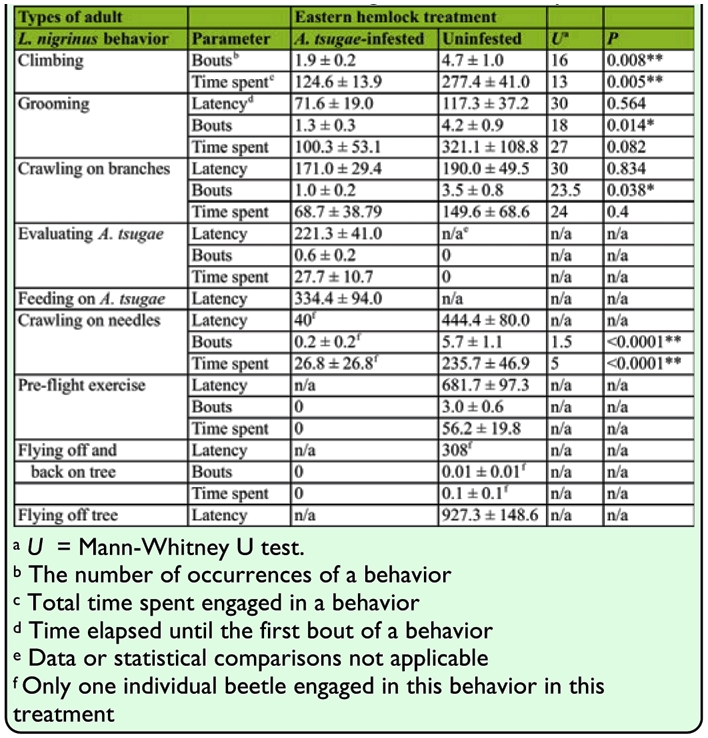
The number of bouts, latency and time spent (in seconds) by newly emerged *Laricobius nigrinus* adults engaged in nine prey searching behaviors on either *Adelges tsugae*-infested or uninfested eastern hemlock seedlings in the laboratory.

Post-emergence flight from the forest floor is likely but was not observed in the visual cue bioassay probably due to the stems being in close proximity to the beetles. In the mass-rearing laboratory, beetles fly readily for a period following emergence in the fall but appear to be less inclined to do so later in the year (D. Mausel, personal observations). Likewise, *L. erichsonii* appears to be capable of long-distance flight and was observed landing at the base of trees (Franz 1958). Whether flying or crawling to a tree, vision provides beetles with a precise direction and location of trees at close range relative to olfaction. Once a tree is found, the arboreal *L. nigrinus* beetles proceed to search for *A. tsugae*. Beetle climbing, crawling on branches, and crawling on needles in the prey searching behavior bioassay are consistent with “extensive searching” described for other aphidophagous predators in the absence of prey (Bell 1990). Crawling on needles while waving the head, antennae, and front tarsi in the air was especially common on uninfested trees, as described in the Coccinellidae (Flowers et al. 2007), Syrphidae, and Chrysopidae (Bell 1991). Generally, these behaviors are used by insects to provide various mechanosensory and olfactory information about their environment when searching (Bell 1990). *Laricobius nigrinus* self-grooming was common on both infested and uninfested trees and is done to clean and optimize the function of sensory organs. The frequent bouts and time spent grooming in the absence of prey was likely done to optimize their senses and re-evaluate the environment before they risked flight dispersal.

In the prey searching behavior bioassay, “evaluating *A. tsugae*” on infested trees is analogous to “local convoluted search” (Jander 1975) and “intensive searching,” described in another *L. nigrinus* study. Flowers et al. (2007) found that this behavior was more common at night, a discovery that suggests that vision is not an important cue at close range of *A. tsugae*. When beetles were among *A. tsugae* they crawled slowly, turned frequently, and appeared to antennate and probe the white flocculence with their maxillary and/or labial palps (Video 2). Contact chemoreceptors may be located on the antennae (Broeckling and Salom 2003a), palps, and tarsi that encounter the waxy flocculence and should be studied further. Furthermore, rain and wind dislodge flocculence from previous *A. tsugae* generations onto branches, boles, and the forest floor and could cue beetles that prey may be present nearby. Other close range nonvisual cues, such as olfaction, may be relied upon to locate *A. tsugae*. Mechanosensory cues are doubtful, because *A. tsugae* is sessile, although nymphs and adults fidget slightly when disturbed.

The primary finding of this study is that *L. nigrinus* searching behavior, like most species, is related to food abundance. Beetles did not fly when *A. tsugae* is abundant and flew readily when *A. tsugae* was absent. This dispersal is consistent with field studies that show a positive correlation between *A. tsugae* density and adult beetle density (Mausel 2007), resulting in beetle aggregations on dense prey patches. Visual cues play a role in their searching behavior and could potentially be exploited for trapping purposes. For example, a bole trap (Hanula and Kirsten 1996) may be effective at capturing newly emerged adults in the field if they climb up tree stems to the canopy.

**Video 1.**
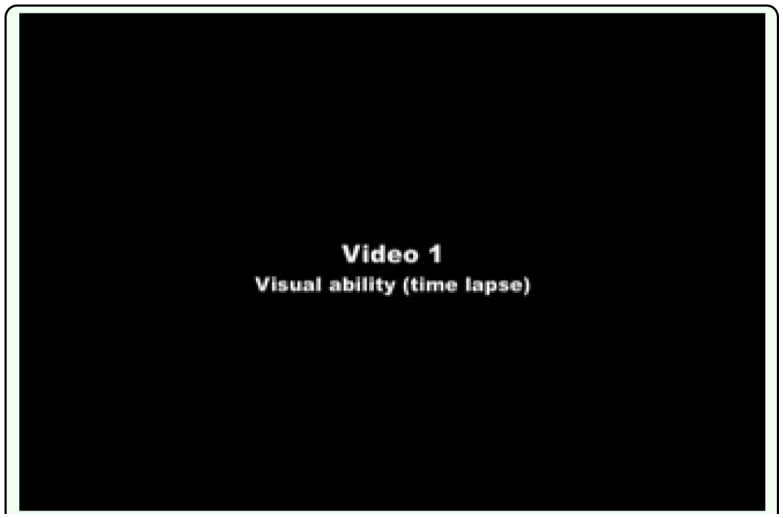
Visual ability. Click image to view video. Download Video

**Video 2.**
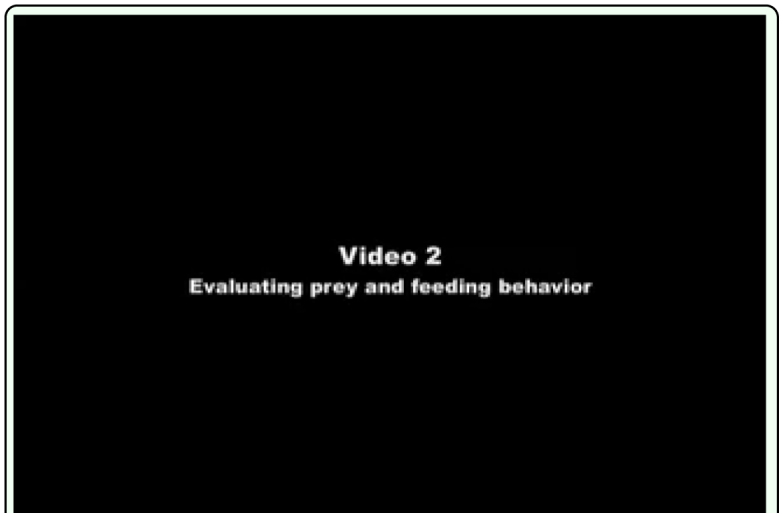
Evaluating prey and feeding behavior. Click image to view video. Download Video

**Video 3.**
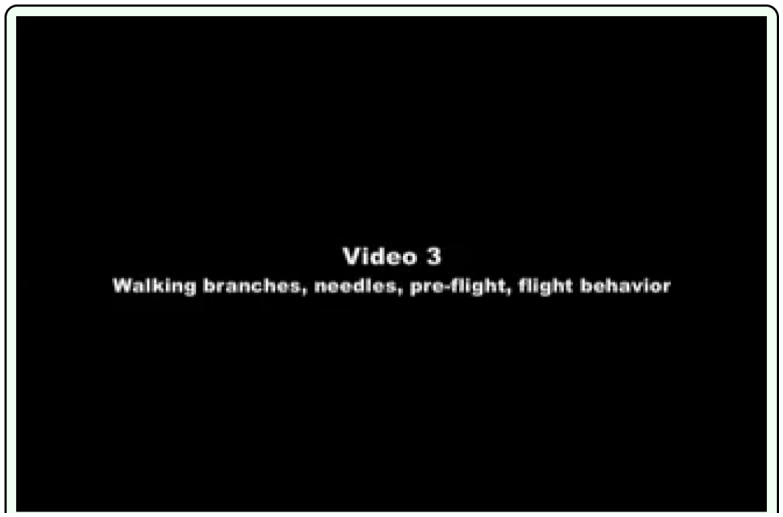
Walking branches, needles, pre-flight, and flight behavior. Click image to view video. Download Video
